# The Critical Role of βPdZn Alloy in Pd/ZnO Catalysts for the Hydrogenation of Carbon Dioxide to Methanol

**DOI:** 10.1021/acscatal.2c00552

**Published:** 2022-04-20

**Authors:** Michael Bowker, Naomi Lawes, Isla Gow, James Hayward, Jonathan Ruiz Esquius, Nia Richards, Louise R. Smith, Thomas J. A. Slater, Thomas E Davies, Nicholas F. Dummer, Lara Kabalan, Andrew Logsdail, Richard C. Catlow, Stuart Taylor, Graham J Hutchings

**Affiliations:** † Cardiff Catalysis Institute, School of Chemistry, Cardiff University, Cardiff CF10 3AT, United Kingdom; ‡ Catalyst Hub, RCAH, Rutherford Appleton Lab, Harwell, Oxford, Didcot OX11 0QX, United Kingdom; § Max Planck-Cardiff Centre on the Fundamentals of Heterogeneous Catalysis FUNCAT, Cardiff Catalysis Institute, School of Chemistry, Cardiff University, Main Building, Park Place, Cardiff CF10 3AT, United Kingdom; ∥ now at: Clean Energy Cluster, International Iberian Nanotechnology Laboratory (INL), Av. Mestre José Veiga, 4715-330 Braga, Portugal; ⊥ Electron Physical Sciences Imaging Centre, Diamond Light Source Ltd., Oxfordshire OX11 0DE, United Kingdom

**Keywords:** methanol synthesis, PdZn alloy, catalysis, carbon dioxide hydrogenation, Pd catalyst, PdZn catalyst, zinc oxide support

## Abstract

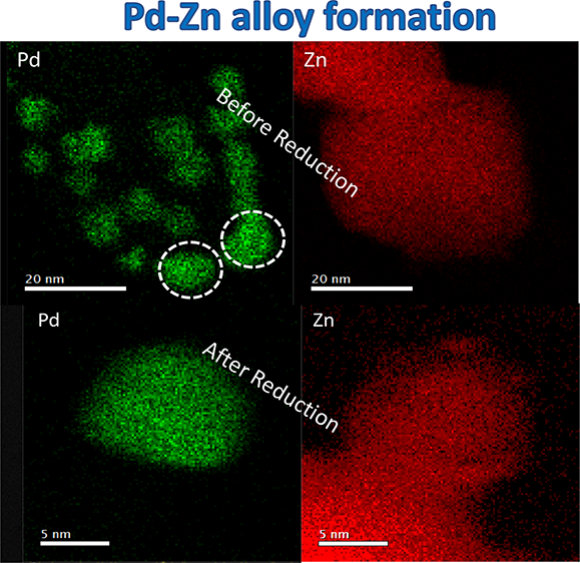

The rise in atmospheric CO_2_ concentration and the concomitant rise in global surface temperature have prompted massive research effort in designing catalytic routes to utilize CO_2_ as a feedstock. Prime among these is the hydrogenation of CO_2_ to make methanol, which is a key commodity chemical intermediate, a hydrogen storage molecule, and a possible future fuel for transport sectors that cannot be electrified. Pd/ZnO has been identified as an effective candidate as a catalyst for this reaction, yet there has been no attempt to gain a fundamental understanding of how this catalyst works and more importantly to establish specific design criteria for CO_2_ hydrogenation catalysts. Here, we show that Pd/ZnO catalysts have the same metal particle composition, irrespective of the different synthesis procedures and types of ZnO used here. We demonstrate that all of these Pd/ZnO catalysts exhibit the same activity trend. In all cases, the β-PdZn 1:1 alloy is produced and dictates the catalysis. This conclusion is further supported by the relationship between conversion and selectivity and their small variation with ZnO surface area in the range 6–80 m^2^g^–1^. Without alloying with Zn, Pd is a reverse water-gas shift catalyst and when supported on alumina and silica is much less active for CO_2_ conversion to methanol than on ZnO. Our approach is applicable to the discovery and design of improved catalysts for CO_2_ hydrogenation and will aid future catalyst discovery.

## Introduction

With the acknowledged effect of CO_2_ on the Earth’s atmosphere in terms of global warming and from the fact that most of this extra CO_2_ (now 414 ppm compared with the historical value of 270 ppm) is derived from fossil fuel burning, there is an urgent need to find non-fossil fuel sources of fuels and chemicals. The main route to clean, green chemistry is to utilize renewable energy to generate electricity, which can be used for various chemical transformations but particularly for large-volume hydrogen production by electrolysis.^1,2^ This paper is concerned with one route to further valorisation of that hydrogen, which is to make methanol by direct hydrogenation of CO_2_ (eq 1). In this situation, the CO_2_ could derive from sources of pollution such as steel and cement production and coal-fired power stations.^3,4^

There are a number of approaches to the understanding of methanol synthesis from CO_2_ and H_2_, both at a fundamental level and from an empirical approach. Scientific progression has historically benefitted from empirical approaches, but new opportunities are now arising in theory- and data-led methodologies that provide chemical accuracy understanding of fundamental reaction processes.^5,6^ Industrially used catalysts (based on Cu/ZnO/Al_2_O_3_, denoted CZA) were developed by incorporating skill and practical know-how in the art of catalyst production.^7,8^ There is some evidence that in the presence of water, CZA catalysts have rather less stability^9,10^ than in the normal, anhydrous methanol synthesis reaction (eq 2).
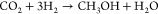
1
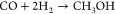
2
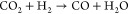
3

Hence, there is a need to explore other catalysts in a search for better activity/selectivity performance and better longevity in the presence of significant water levels. In this paper, we present detailed results for the behavior of PdZn alloy catalysts prepared by a range of different synthetic procedures. As we showed previously,^11^ Pd/ZnO is a good reverse water-gas shift (RWGS) catalyst (eq 3), but if it is pre-reduced above 300 °C, it makes a very good methanol synthesis catalyst by reaction 1. The change in reactivity is because the β-PdZn 1:1 alloy is produced upon reduction, which appears to suppress the RWGS activity and enhance the methanol synthesis activity. There are several other publications relating to this alloy system for methanol synthesis.^12−22^ The objective of the current work is to investigate the role of different preparation methods in forming the PdZn alloy and to determine which types of synthetic procedures give the best performance for methanol synthesis.

## Results and Discussion

**Figure 1:**
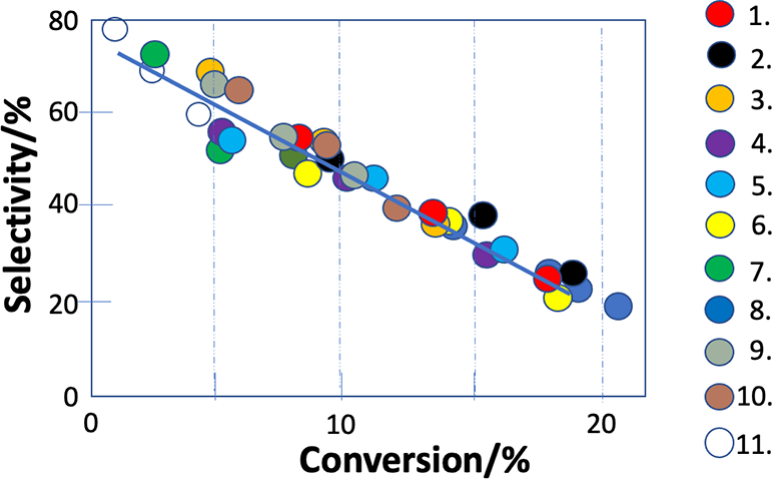
Methanol selectivity–CO_2_ conversion data for a variety of Pd-ZnO catalysts that are listed in Table 1. There are three data points for each catalyst taken at reaction temperatures of 230, 250, and 270 °C.

We have measured the reactivity of a wide range of Pd-ZnO catalysts by use of a high throughput reactor (described in the Methods section below). The catalysts were prepared using a range of ZnO supports, including two different commercial samples, some materials of higher surface area made by the modified Farag method^23^ (see Supplementary Information), and even higher area ZnO made by precipitation. The Pd was then added to the surface of the ZnO by chemical vapor impregnation (CVI), impregnation, deposition-precipitation or sol immobilization. Figure 1 summarizes the methanol selectivity and CO_2_ conversion results for these catalysts, while Table 1 gives the details of the various types of catalyst used (full details are provided in the Supplementary Information).

**Table 1:** Details of the Catalysts Used in Figure 1

catalyst	support (surface area/m^2^g^–1^)	method of synthesis
1. 5wt%Pd/ZnO^b^	Commercial ZnO 1^a^ (15)	Pd by CVI
2. 5wt%Pd/ZnO	Commercial ZnO 1^a^ (15)	Pd by deposition precipitation
3. 3wt%Pd/ZnO	Commercial ZnO 2^a^ (6)	Pd by CVI
4. 3wt%Pd/ZnO	Modified Farag ZnO (MFZ) (26)	Pd by CVI
5. 3wt%Pd/ZnO	MFZ (26)	Co-precipitation
6. 5wt%Pd/ZnO	MFZ (26)	Pd by CVI
7. 1wt%Pd/ZnO	MFZ (26)	Pd by CVI
8. 15wt%Pd/ZnO	MFZ (26)	Pd by CVI
9. 3%wtPdZn/ZnO	MFZ (26)	Pd and Zn by CVI
10. 3%wtPdZn/ZnO	Commercial ZnO 2^a^ (6)	Pd and Zn by CVI
11. 5%wtPd/ZnO	Commercial ZnO 1^a^ (15)	Sol immobilization from Pd(NO_3_)_2_

a 1 is Sigma Aldrich ZnO, 2 is Acros ZnO.

b Note that for catalyst 1, this was measured four times, with two different batches, and the data in Figure 1 is the average, as described later in the text.

A clear linear relationship between selectivity and conversion is immediately apparent for all these catalysts, which might be considered surprising since it includes several very different methods of preparation. The relationship appears to be a characteristic for this catalyst system, that is, for PdZn nanoparticles supported on ZnO. The formation of the PdZn alloy after reduction of the Pd/ZnO system has been shown previously by us^11,24,25^ and others^12−22^ for certain specific preparations of the catalyst. Some other types of preparation did not result in formation of the PdZn alloy, and then these did not perform in this way. As a base, Pd/γ-alumina showed poor activity and selectivity to methanol and was a reverse water-gas shift catalyst (see Table S1). For comparison purposes, the total CO_2_ turnover frequency for the catalyst sample 1 at 250 °C is 5.5 × 10^–3^ molecules/site/s and that to methanol is 2.2 × 10^–3^ molecules/site/s (see Supplementary Information, Section 3).

**Figure 2:**
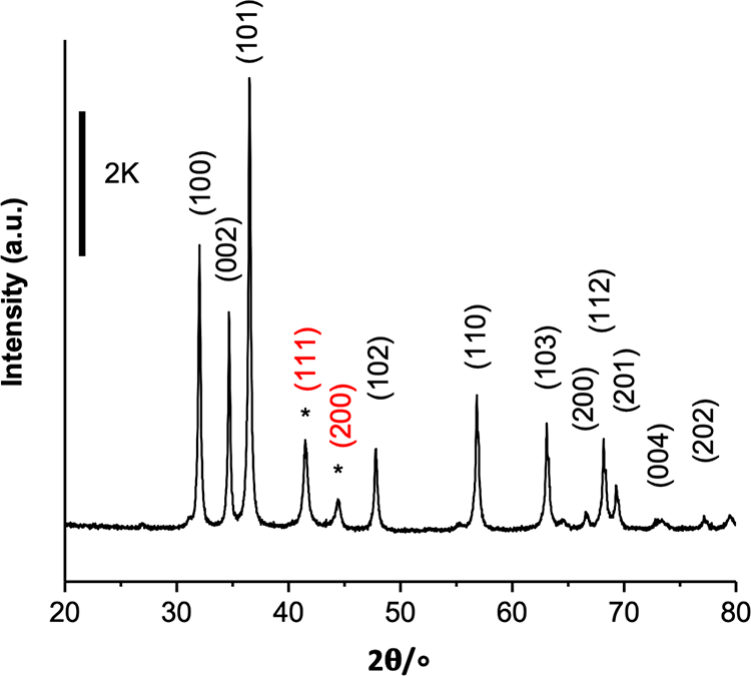
XRD of the 15 wt % Pd/ZnO catalyst after reduction at 400 °C, reflections of ZnO can be indexed to the standard JCPDS-36-1451. The catalyst was prepared by chemical vapor impregnation onto Sigma Aldrich ZnO (ZnO 1). *Reflections of β-PdZn crystallites.

**Figure 3:**
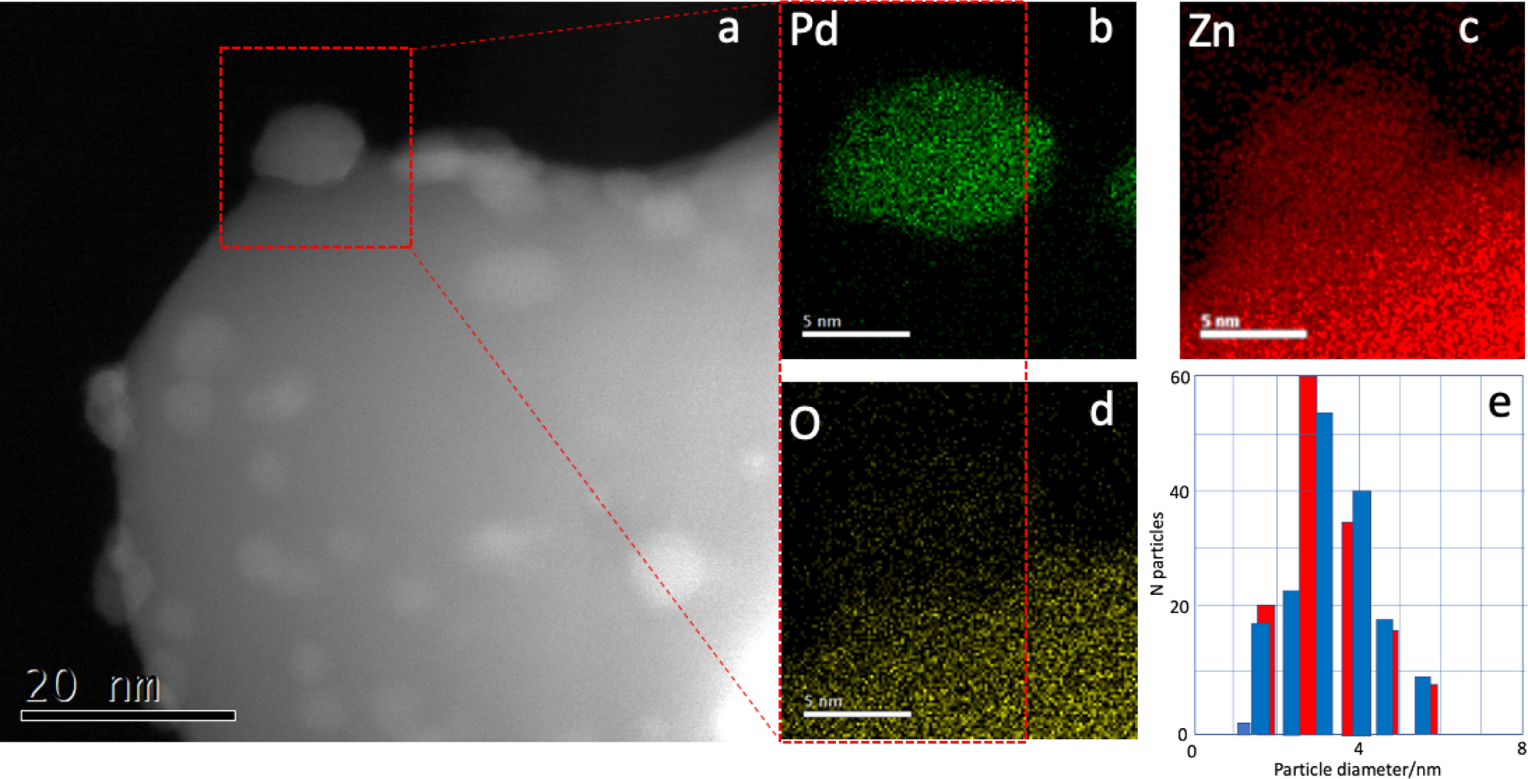
(a) Annular dark-field STEM image of 5% Pd/ZnO made by CVI of Pd onto the commercial ZnO 1 (catalyst 1) after reduction in hydrogen at 1 bar pressure at 400 °C. The highlighted particle has been mapped by EDX spectroscopy to show the formation of the PdZn alloy (images b– d). (e) Particle size distribution from larger area maps. Red bars, for the reduced 5%Pd/ZnO (catalyst 1), with a total particle count of 140. The blue bars are for the post-reaction catalyst run for 96 h between 230 and 270 °C, with a total particle count of 160.

**Figure 4:**
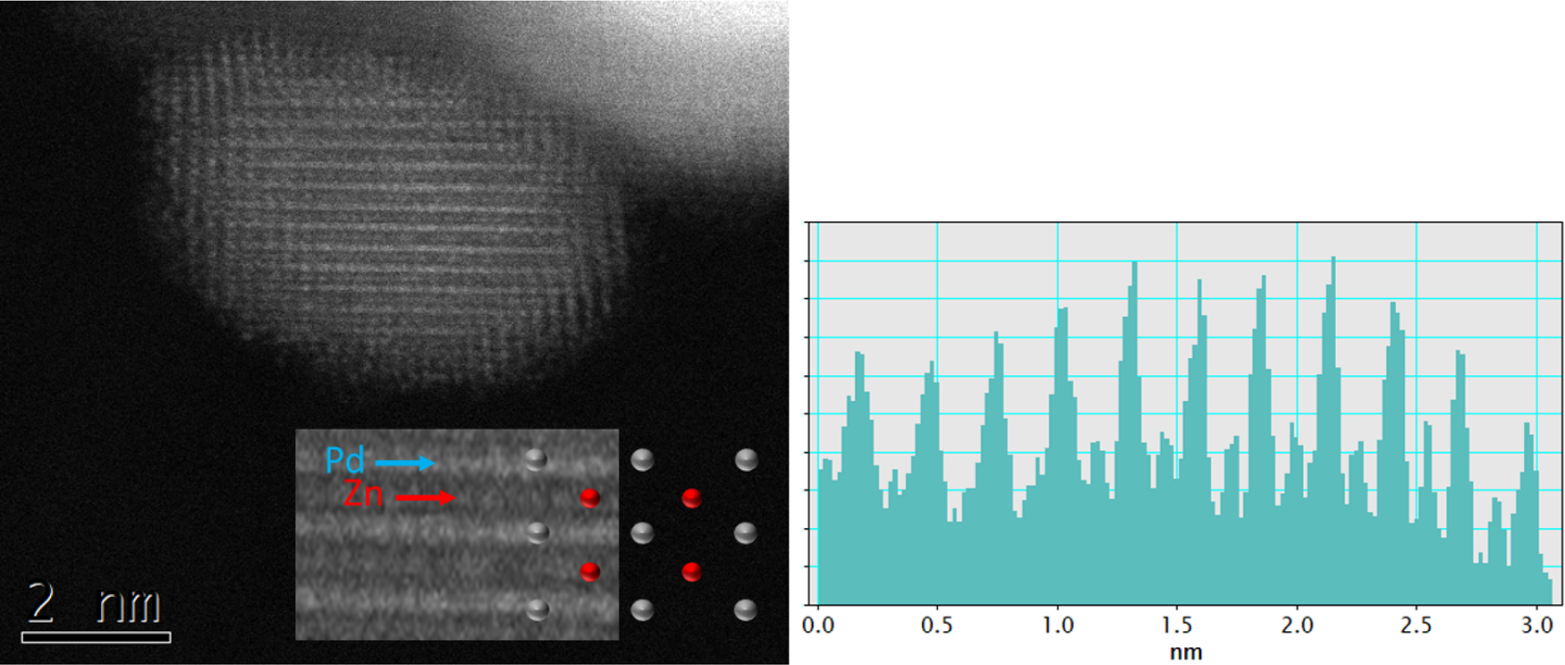
Lattice imaging of the structure of the PdZn particles in sample 1, 5% Pd/ZnO1, after reduction, using HAADF STEM, together with a model showing the [0–10] projection of the top layer. A line scan profile of the linear features in the image is shown on the right.

Figure 2 shows that the β-PdZn alloy has formed by reduction using XRD. The alloy has shifted diffraction peaks at 41.2° and ∼44° compared with monometallic Pd at ∼40°. We show one example here for the 15% loaded catalyst since the PdZn peaks are clearest at this high loading, but XRD for a number of other catalysts is given in Figure S1. Further, energy-dispersive X-ray (EDX) mapping in a scanning transmission electron microscope (STEM) (Figure 3 and Figure S2) quite clearly shows the formation of the alloy on catalyst 1, whereas prior to reduction, STEM-EDX shows that the catalyst comprises of PdO particles on ZnO (Figure S3). *In situ* XRD shows that some of the PdZn has formed by around 300 °C.^11,24,25^ Using HAADF STEM, the lattice can be resolved, Figure 4 and Figure S2, and is the same as that reported by Nowicka *et al*., who obtained atomic resolution images of the lattice showing the good order in the particle lattice;^26^ the lattice projection of the [0–10] plane is shown in the figure. Note that MacLeod *et al.* deposited Zn onto a Pd(111) single crystal and form a (1 × 2) 1:1 surface alloy by substitution of Zn into the Pd lattice.^27^ The PdZn particles have a narrow size distribution (Figure 3e), ranging from around 1.5 nm to around 6 nm, with most around 3.5 nm, and there is, within experimental error, no change in size distribution after reaction. Images are also shown in Figure S4 for sample 6, which shows similar alloying within the particle, even after reduction at as low as 200 °C for 1 h. All of these results show that CVI is an excellent method for making small, stable metal nanoparticles.

First-principles parameterized calculations by Kozlov *et al*(28) show alloy formation for PdAu, PdCu, PdAg, and PdZn; in contrast to the other alloys, PdZn forms a uniform alloy with no particular elemental segregation and the same ratio (1:1) in the surface as in the bulk, at least under the vacuum conditions of the simulation. Note also that Peterson *et al.*(43) showed that aerosol-synthesized PdZn particles were the perfect 1:1 alloy, with remarkably low defects: to quote that paper “detailed structural analysis shows that this material contains little or no vacancies and minimal Pd/Zn disorder”, as was found in the theory work of Kozlov *et al*.

It seems that no matter which preparation method is used, we obtain essentially the same PdZn alloy, at least for the cases reported here, though it is likely that not all preparation procedures will work. They certainly will not if the alloy nanoparticles are not produced.

**Figure 5:**
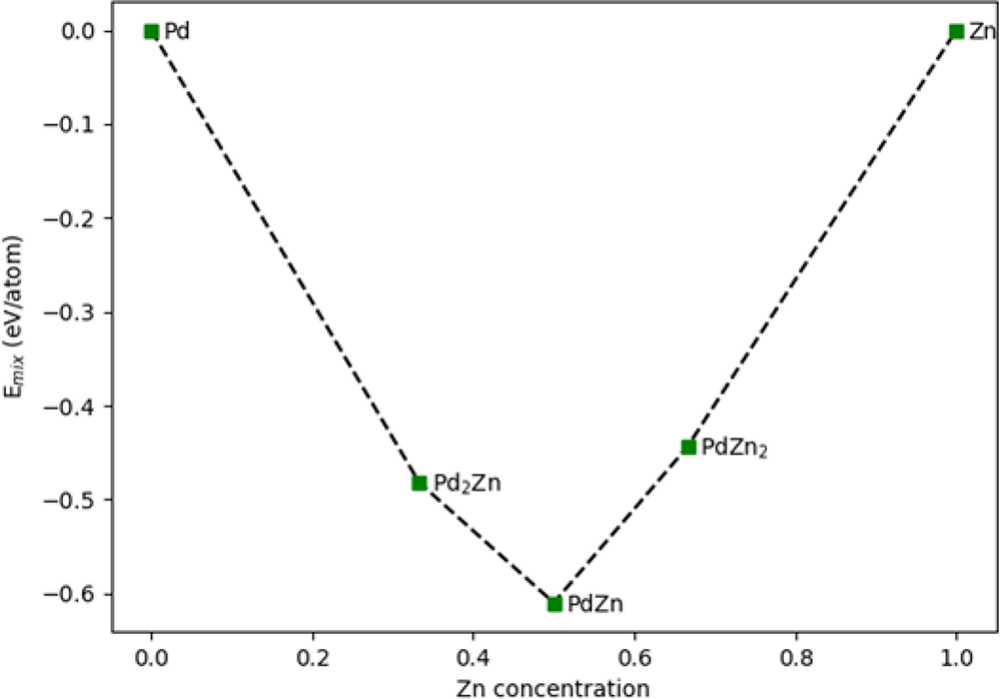
Calculated mixing energy (*E*_mix_) of Pd and Zn, showing a minimum for the (1:1) compositional alloy with the β-PdZn structure.

First-principles density functional theory (DFT) calculations were used to determine the mixing energy per atom (*E*_mix_) for Pd_2_Zn, PdZn, and Zn_2_Pd using the following equation:
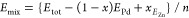
4where *E*_tot_ is the calculated total energy; *E*_Pd_ and *E*_Zn_ are the energies of the respective Pd and Zn bulk systems, with the same quantity of atoms, *n*; and *x* the Zn concentration. *E*_mix_ for PdZn is −0.61 eV/atom compared to the −0.482 eV/atom for Pd_2_Zn and −0.48 eV/atom for PdZn_2_. These results show that PdZn 1:1 has the highest heat of mixing in the phase diagram (Figure 5). Comparing *E*_mix_ of PdZn with other binary alloys, PdZn is found to have a higher *E*_mix_ than AuPd and CuPd, which have *E*_mix_ equal to −0.10 eV/atom and −0.17 eV/atom, respectively. The results for CuPd and AuPd agree with the calculations of Seko *et al.*(29) It is noted that Peng *et al.* also found minima in free energy for AuPd and AuAg at the 1:1 ratio, but it is not so for CuAu alloys.^30^

Reconsidering the catalyst reactivity, our original paper in 2016^11^ showed that a catalyst prepared by incipient wetness was not selective for methanol and had lower total activity. It was considered that this was due to the presence of Cl in the preparation, some of which is still present in the final catalyst, and due to the formation of much larger metal nanoparticles, also due to the presence of Cl. When the catalyst is made from a nitrate precursor, the performance is in line with the catalysts above, although the activity is not very high (5% conversion at 270 °C). Further, for catalyst 2, where deposition precipitation was used, even though it uses PdCl_2_ as the precursor, the urea reductant removes the effect of Cl and its presence at the surface of the final catalyst. This is because it is not the chloride that is deposited on the surface, but the hydroxide of Pd, while the chloride is left in solution.^31^

We have also investigated two different batches of Pd/ZnO, prepared at different times by the same CVI method, tested in separate runs of the reactor and in different reactor beds, to give us some idea of the uncertainty in the determinations of selectivity and conversion, and the graph for those data is given in the Supplementary Information (Figure S5). The data show a mean deviation in conversion of ±0.6% and in selectivity of ±3%. For one run with 12 tubes of the same catalyst from the same batch, the mean deviation in conversion was ±0.3% and in selectivity was ±0.2%. For individual measurements in that case, the deviations can be ±0.6% in conversion and in selectivity. More details about this are given in the Supplementary Information.

If we vary the weight loading of Pd (Figure 1, catalysts 4 and 6–8), the resulting data are also close to the linear relationship, but with varying activity, and the same applies to total catalyst weight loading variation (see Figure S6).

**Figure 6:**
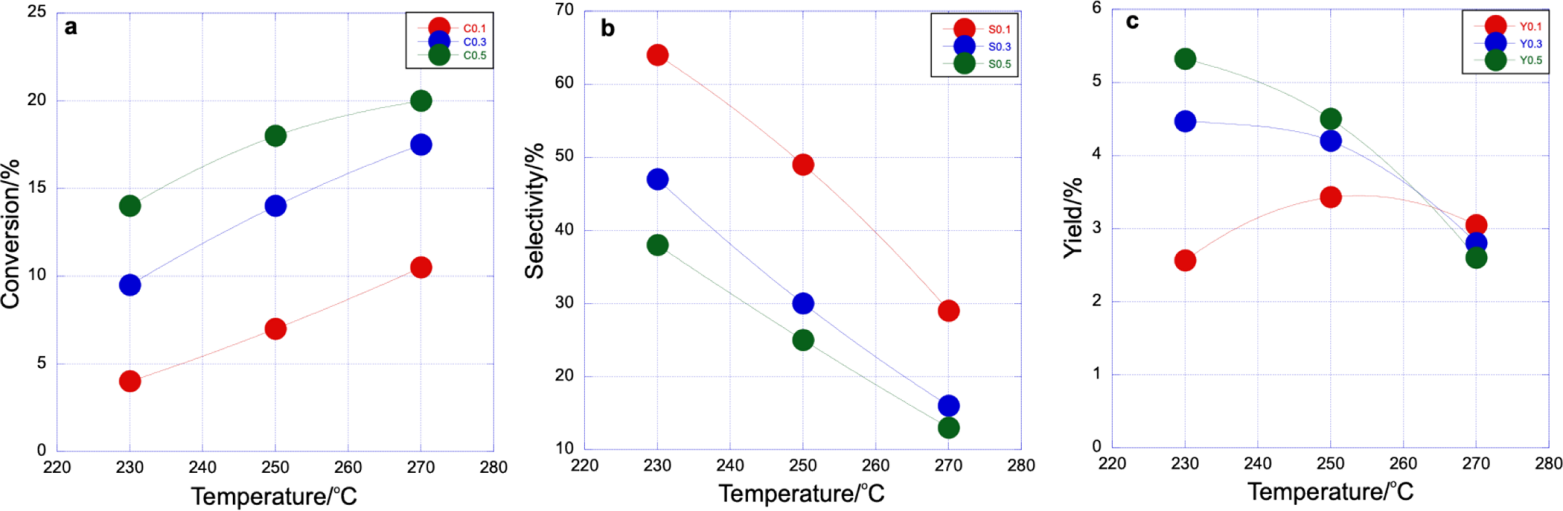
Total weight loading dependence of the CO_2_ conversion, methanol selectivity, and yield as a function of reaction temperature for catalyst 8 with 15 wt % loading of Pd. Red circles, 0.1 g; blue, 0.3 g; green, 0.5 g.

The interdependence of selectivity and conversion can be expressed by the following linear relationship
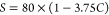
5where *S* here represents the selectivity and *C* is the conversion, both as percentages. The zero-conversion limit (80%) implies that there is a branching between two different reactions, one producing CO and one producing methanol, with a branching ratio of 0.8 for methanol by linear extrapolation to zero conversion. However, the branching ratio changes with conversion, probably due to the acceleration of the RWGS with increasing water presence in the gas stream. However, as the total reactor weight loading increases (effectively bed length dependence), as shown in Figure 6 and Figure S7, the rate to methanol decreases down the bed, while that to CO increases. Here, the initial selectivity is determined by the branching ratio, but as the reaction proceeds down the bed, the methanol yield plateaus, Figure 6. This is probably due to the presence of increasing amounts of water formed down the bed (from both methanol and CO synthesis), which encourages the reverse of methanol synthesis (eq 1).

The linearity of the selectivity–conversion data in Figure 1 implies that we have two parallel reactions, reactions 1 and 3 above, with the latter being the RWGS. The results may imply a surface intermediate, which is common to both reactions and has a certain probability of going to one product or the other. The extrapolation to zero conversion from Figure 1 and eq 5 shows that this favorability is about 80% toward methanol synthesis but decreases in all cases as temperature increases. Using the three temperature data points, the apparent activation energy for methanol production is near zero, whereas for CO, it is around 75 kJ mol^–1^ (Figure S8), which is very similar to the value reported by Liao *et al.*(17) The common intermediate is likely to be the formate species^11,17,25,32−34^ and that intermediate has been shown by infrared spectroscopy to be present during synthesis.^17,34^ Liao *et al.*(17) propose that the role of Zn is to shift the main intermediates present from COOH, which is proposed for the RWGS and mainly produces CO on pure Pd, to the formate, which favors methanol, when the alloy is formed. It could be that the reverse of this conversion (HCOO to COOH) is enhanced in the presence of water.

Although all the catalysts in Figure 1 show the PdZn characteristic linear relationship, that does not mean they all perform in exactly the same way. For instance, for Pd deposited onto ZnO, by CVI, the CO_2_ conversion for 15% Pd weight loading is around 14% at 230 °C, with a selectivity of 35% to methanol, whereas the values for conversion and selectivity at 1% Pd loading are 2.5 and 72%, respectively, showing a strong dependence of performance on Pd loading.

We note that the line of Figure 1 seems to be a characteristic for PdZn/ZnO, and that other groups of catalysts (e.g., Pd/TiO2, Cu/ZnO, Cu/ZrO2) differ from those in Figure 1 (to be reported when completed in a later publication). The question remains, however, why do all of these PdZn/ZnO catalysts display this same characteristic? It may simply be a consequence of the electronic structure of β-PdZn and that this alloy is always formed in these experiments. Pd metal itself is a transition element with a d band crossing the Fermi level and one that is generally considered to be nearly full (bulk Pd for instance is quoted to have an average 3d filling of at least 9^35^). When Pd is alloyed with s band metals, with a low density of state at the Fermi level, there tends to be some charge transfer, both intra- and interatomically with the Pd,^36^ leading to d state filling and a shift of the d band away from the Fermi level. In simplistic terms, it may be that Pd becomes more like Cu. Cu is the basis of commercial catalysts for methanol synthesis but with high levels of metal present – usually *ca.* 50% loading. Regarding PdZn, theoretical calculations also show that the 1:1 β-alloy phase is the most stable phase to be found since it has the maximum exothermic heat of mixing (Figure 5), and Kozlov *et al*(28) showed a homogeneous alloy for PdZn with no preferential surface segregation of either element.

**Figure 7:**
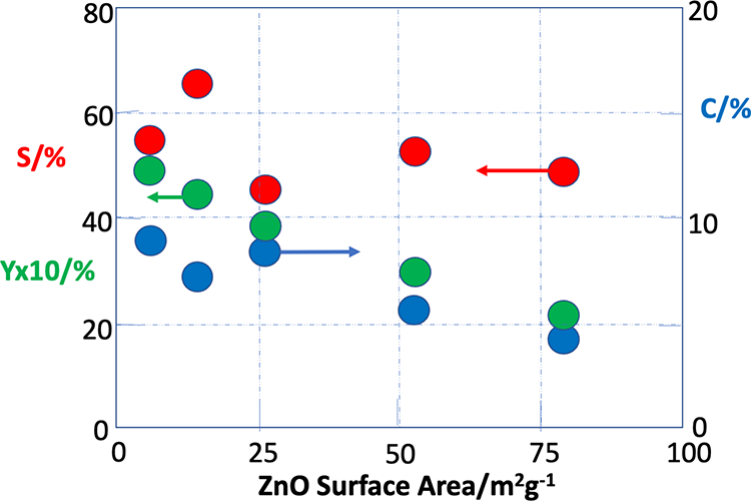
Dependence of reaction properties at 230 °C on ZnO surface area. All catalysts are 5 wt % Pd/ZnO, made by CVI of the Pd, except the data at 55 m^2^g^–1^, which is for a Pd/ZnO catalyst doped onto the surface of P25 TiO_2_ in order to have an intermediate surface area. In that case, the titania is covered by ZnO. Blue circles are for CO_2_ conversion, red are for methanol selectivity, and green are for methanol yield (×10).

Finally, a further unusual feature of this system is that the performance is near-independent of the surface area of the support. Figure 7 shows the dependence of selectivity and conversion upon surface area of the various ZnO supports (Table 2) for samples prepared by the same Pd deposition method, namely CVI. The surface area varies by more than an order of magnitude, and yet there is surprisingly little change in performance from low to high area. There is little clear loss of selectivity as surface area goes up, but there is some decrease in conversion, and this is accompanied by about a two-fold decrease in yield. These data further support the idea that, provided the PdZn is supported on ZnO, the performance is dictated by the PdZn nanoparticles themselves. Nonetheless, it would be anticipated that there would be at least some dependence of PdZn particle size upon the method of deposition of the Pd and the size of the support particles. It was anticipated that increasing the support area would improve the catalysts, but the reverse was observed – there was a slightly negative dependence of yield on support surface area.

**Table 2:** Surface Area of the Various ZnO Supports Used^a^

support	surface area /m^2^ g^–1^
Acros ZnO	6
Sigma Aldrich ZnO	15
Farag method ZnO	26
Precipitation ZnO^33^	78
P25 TiO_2_	55

a Note that the final support in this list was made by depositing Pd and Zn (in a 1:10 ratio) onto titania by CVI in order to have an intermediate surface area. In that case, the titania is covered by ZnO. Characterization of that material is given elsewhere.^24^

The near independence of these properties over this range of support area remains puzzling. One explanation is that the PdZn particle size is near-independent of the support area for the CVI method. As shown above, the average particle diameter is around 4 nm for the Pd/ZnO catalyst 1, but it is also similar for a ZnO support of 102 m^2^ g^–1^ surface area. So, it seems that the CVI method is effective at producing similarly sized particles no matter what the support area within this range. It also must be noted that in our earlier paper, using sol immobilization, the performance varied little (selectivity falls from 60 to 55%, while conversion falls from 8.1 to 6.5%) for a change in average PdZn particle size of a factor of two from *ca.* 2.7 to 5.4 nm.^11^

## Conclusions

We have shown that a wide variety of methods are effective for producing PdZn nanoparticles supported on ZnO. The β-PdZn 1:1 alloy is formed in all cases and theory confirms that this alloy has the most exothermic heat of mixing, thus favoring that alloy. The same observations are made for a variety of ZnO surfaces, synthesized in this work or bought commercially. Two surprising features of this system are shown: first, there is a linear relationship between selectivity and conversion for the PdZn materials, which applies for different preparation methods and different metal loadings; second, the relationship between support surface area and selectivity/conversion is very weak (little variation) for more than an order of magnitude change in ZnO surface area.

## Experimental Section

### Catalyst Synthesis

a) ZnO support. We used a variety of ZnO supports. Two of these were commercial ones – from Acros (ZnO 2, 99.5 + %) and Sigma Aldrich (ZnO 1, 99.9%). We also synthesized ZnO by several methods, the details of which are given in the Supplementary Information. One is a modification of the Farag method,^23^ which is a precipitation method to form a zinc hydroxy carbonate material from zinc acetate and sodium carbonate, followed by filtering, washing, drying, and calcination to 450 °C. This results in the formation of a ZnO of higher area than the commercial ones. An even higher surface area is obtained by the method described by Bowker *et al*.,^37^ which is a precipitation method using zinc nitrate and sodium carbonate, forming a zinc carbonate precipitate, which is filtered and washed, dried, and calcined at 300 °C.
b) Pd addition. Pd was added to the catalyst in a number of ways. The most usual method we used was CVI (chemical vapor impregnation), in which the ZnO was mixed with Pd(acac)_2_, heated under vacuum to 133 °C, and subsequently calcined at 500 °C. The method is described in more detail in the Experimental section. Some catalysts were made in this way but using CVI for both Zn(acac)_2_ and Pd(acac)_2_ onto a ZnO support. Other catalysts were made by deposition precipitation, co-precipitation, and sol immobilization, as described in the Supplementary Information.

#### Catalyst Analysis

The materials were analyzed by XRD, TEM, SEM, and BET measurement, as described in the Supplementary Information.

#### Catalyst Testing

The catalytic performance was determined in a parallel 16 bed high throughput catalytic reactor, designed and manufactured by Integrated Lab Solutions (ILS). The reactor is operated using Integrated Workflow Manager, based on LabVIEW and automated using Siemens SIMATIC S7-1500 and Siemens Win CC SCADA Software. The high throughput reactor is of a fixed bed, continuous flow design, and all reactor beds see the same reaction conditions due to the setup of the system configuration, with all the gases fed through a capillary distribution system. The pressure is controlled in each bed using an equilibar, and the temperature is controlled by thermocouples located in the heating block surrounding each set of four reactor tubes. The catalyst (usually 0.5 g pelleted to 425–600 μm) was placed in a stainless steel reactor tube with an internal diameter of 4.57 mm. The catalysts were placed in all beds, apart from beds 1, 5, 9, and 13, and these were filled with only silicon carbide (F24, 750 microns). The catalyst was supported on a bed of silicon carbide (F24, 750 μm) to ensure it was centered in the isothermal heating zone. The catalyst was mixed with F80 silicon carbide (0.75 g), which has a particle size of 190 μm, to aid dispersion and improve heat transfer across the catalyst bed. Prior to the reaction, the catalysts were pre-reduced in a flow of 5% H_2_/N_2_ (40 mL min^–1^ per reactor bed) for 1 h at 400 °C (5 °C min^–1^ ramp rate) under atmospheric pressure. Once the reduction was complete, the reactors were cooled to 125 °C and the gas composition was switched to the reaction feed (CO_2_:H_2_:Ar:N_2_ 22:54:5:19%). A purge feed of nitrogen, equivalent flow to the reaction feed, is used to ensure that no product buildup is observed in the downstream lines. The pressure was increased to 20 bar using the gas feed pressure over the equilibars. The flow rate was 31.3 mL/min at atmospheric pressure. The reactions were conducted at 230, 250, and 270 °C, with the downstream oven set to 120 °C to stop any product condensing in the reactor lines. The gas products were analyzed *via* online gas chromatography using an Agilent 7890B system with two flame ionization detectors (FID) and a TCD. Argon was used as an internal standard. Four injections were taken at each temperature per reactor bed, and sampling of the products was achieved with a Vici stream selection valve to switch between the beds. It took *ca*. 8 h to sample all beds; therefore, it took 32 h to complete one reaction temperature. CO_2_ conversion was calculated by the change in moles of CO_2_ compared to calibration runs at 125 °C for each bed. In all cases, methanol, methane, and CO were the only products observed. The carbon balance was calculated using the sum of carbon products and feed divided by the blank carbon value. After the reaction, the reactor was depressurized and left to cool under flowing nitrogen (50 mL min^–1^).

### Calculations of Alloy Heats of Mixing

All calculations have been performed with the “Fritz Haber Institute ab initio molecular simulations” (FHI-aims) all-electron full potential software package.^38^ Calculations were performed with the mBEEF exchange correlation density functional^39^ from the LibXC DF density functional library,^40^ using a light basis set and a **k**-grid density of (0.019 × 2π) Å^–1^.

The self-consistent field (SCF) cycle was deemed converged when the changes in total energy and density were less than 1e^–6^ eV and 1e^–6^ e a_0_,^3^ respectively. Throughout, a spin-paired configuration has been used with scalar relativity included via the atomic zero-order regular approximation (ZORA).^41^ For geometry optimizations, convergence was deemed complete when forces on all unconstrained atoms were less than 0.01 eV/Å.

Lattice parameters for Cu, Au, Pd, and Zn pure structures were taken from Janthon *et al*.^42^ After unit cell optimization with relaxation of lattice parameters, angles, and atom positions, Pd, calculated in a face-centered cubic (FCC) structure, has a lattice parameter of 3.893 Å (*vs* 3.909 Å reported by Janthon *et al.*(42)); Cu has an optimized FCC lattice parameter of 3.575 Å (*vs* 3.594 Å);^5^ Au has an optimized FCC structure with an optimized lattice parameter equal to 4.092 Å (*vs* 4.030 Å); and Zn has an optimized hexagonal closed packed (HCP) lattice with *a* = 2.662 Å and *c* = 4.955 Å (*vs* 2.665 and 4.955 Å). The cohesive energy values for the pure metals have been calculated and are 3.58, 3.42, 3.53, and 1.33 eV for Pd, Au, Cu, and Zn respectively, which agree with the experimental values reasonably well: (3.90, 3.81, 3.48, and 1.35 eV for Pd, Au, Cu, and Zn, respectively).

For the PdZn crystallographic phases, the unit cells derived from experimental XRD have been used as the starting configurations for optimization. For PdZn, the β phase has been calculated as *a* = *b* = 2.890 Å and *c* = 3.330 (*vs a* = *b* = 2.889 Å and *c* = 3.346 Å, given by Peterson *et al.*);^43^ for Pd_2_Zn, *a* = 5.327 Å, *b* = 4.142 Å, *c* = 7.742 Å (*vs a =* 5.350 Å, *b* = 4.140 Å, *c* = 7.650 Å, given by Stadelmair *et al.*;^44^ and for Zn_2_Pd, *a* = 5.238 Å, *b* = 5.239 Å, *c* = 12.172 Å (*vs a* = 5.327 Å, *b* = 5.327 Å, *c* = 12.235 Å given by Alasafi *et al.*)^45^ For AuPd and CuPd, the conventional body-centred cubic unit cell was used as the starting point, with *a = b = c* = 3.970 Å for AuPd (*vs* 3.980 Å given by Venudhar *et al.*)^46^ and *a = b = c* = 2.939 Å for CuPd (*vs* 3.063 Å given by Yamauchi *et al.*).^47^ In all cases, α *=* β *=* δ = 90°.

## Supporting Information Available

More detailed information about catalyst preparation (section 1) and characterization (section 2, Figures S1–S4) and further reactor data (section 3, Figures S5–S7) and Arrhenius plots (Figure S8) (PDF)
