# Supplementary material for: The Critical Role of βPdZn Alloy in Pd/ZnO Catalysts for the Hydrogenation of Carbon Dioxide to Methanol
Source: ACS Catal. 2022 Apr 20;12(9):5371–9. doi: 10.1021/acscatal.2c00552 (PMC9087181; doi:10.1021/acscatal.2c00552)
Supplement: Supplementary file 1 — cs2c00552_si_001.pdf [file cs2c00552_si_001.pdf]

Supporting information for

The Critical Role of βPdZn Alloy in Pd/ZnO Catalysts for the Hydrogenation of Carbon Dioxide to Methanol

Michael Bowker[1,2,3*], Naomi Lawes[1,3], Isla Gow[1,3], James Hayward[1], Jonathan Ruiz Esquius[1,4], Nia Richards[1], Louise R. Smith[1,3], Thomas J. A. Slater[5], Thomas E Davies[1], Nicholas F. Dummer[1,3], Lara Kabalan[1], Andrew Logsdail[1], Richard C. Catlow[1,2,3], Stuart Taylor[1,3] and Graham. J Hutchings[1,3]

[1]Cardiff Catalysis Institute, School of Chemistry, Cardiff University, Cardiff CF10 3AT, United Kingdom

[2]Catalyst Hub, RCAH, Rutherford Appleton Lab, Harwell, Oxford, Didcot OX11 0QX, United Kingdom

[3]Max Planck-Cardiff Centre on the Fundamentals of Heterogeneous Catalysis FUNCAT, Cardiff Catalysis Institute, School of Chemistry, Cardiff University, Main Building, Park Place, Cardiff, CF10 3AT, United Kingdom.

[4]now at: Clean Energy Cluster, International Iberian Nanotechnology Laboratory (INL), Av. Mestre José Veiga, 4715-330 Braga, Portugal

[5] Electron Physical Sciences Imaging Centre, Diamond Light Source Ltd., Oxfordshire OX11 0DE, UK

Corresponding author email: bowkerm@cardiff.ac.uk

Experimental

1. Catalyst Preparation

As well as using the Sigma Aldrich and Acros ZnO described in the main text, other forms of higher surface area ZnO were also produced in-house.

1.1. Zinc oxide preparation: Zinc hydroxycarbonate method

$Zn(CH_3CO_2)_2{\cdot}2H_2O$ (≥ 98 %) and $(NH_4)_2CO_3$ (≥ 30 % NH_3 basis) were both obtained from Sigma Aldrich. The chemicals were used as provided and without further purification.

Initially, higher surface area ZnO was prepared in accordance with the method published by Farag *et al.*,[1] with some modifications. Firstly, as described in the original method, stock solutions of $Zn(CH_3CO_2)_2$ and $(NH_4)_2CO_3$ (both 0.5 M) were prepared. A 600 mL glass beaker was charged with $Zn(CH_3CO_2)_2$ (100 mL), then $(NH_4)_2CO_3$ solution (100 mL) was added quickly yet cautiously with vigorous stirring. The mixture was heated in an oil bath to 60 °C and aged for 1 hour, stirring all the time. Once the ageing period was complete, the precipitate was filtered under vacuum, washed with 2 L deionised water and dried in an oven at 110 °C for 16 hours. The zinc hydroxycarbonate was then collected and ground in a pestle and mortar, then calcined under flowing air at 450 °C for 3 hours to give the desired zinc oxide product. The resulting surface area was measured to be 25 m^2/g.

1.2. Zinc oxide preparation: Zinc carbonate method

$Zn(NO_3)_2 \cdot 6H_2O$ (≥ 98 %) was obtained from Sigma Aldrich, and Na_2CO_3 (anhydrous) was obtained from Fischer Scientific. The chemicals were used as provided and without further purification.

Another method was developed to synthesise ZnO with even greater surface area. This combined a method published by Bowker *et al.*[2] and the method used previously from Farag *et al.*[1] Stock solutions of $Zn(NO_3)_2$ and Na_2CO_3 (both 0.5 M) were prepared. In a 600 mL glass beaker, firstly the $Zn(NO_3)_2$ solution (100 mL) was added, then with vigorous stirring the Na_2CO_3 solution (100 mL) was added quickly but carefully. The mixture was heated in an oil bath to 60 °C and maintained for 1 hour with stirring. The precipitate was then filtered under vacuum, washed with plenty of deionised water (2 L), and dried in an oven at 110 °C for 16 hours. The zinc carbonate intermediate was collected, then ground in a pestle and mortar. After calcining for 6 hours at 300 °C under flowing air, the resulting zinc oxide was found to have a surface area of 78 m^2/g.

1.3 Final Catalyst Preparation

1.3.1. Conventional co-precipitation and modified deposition precipitation.

$Pd(NO_3)_2 \cdot 2H_2O$ (40 % Pd basis) was supplied by Sigma Aldrich, and was used as provided and without further purification. Other materials were provided as described above.

The co-precipitation method tested was based on the two ZnO preparations outlined in parts *1.1.* and *1.2.* In each case, once the Zn precursor was charged into the reaction vessel, and $Pd(NO_3)_2$ solution was then added. The precipitating agent, Na_2CO_3, was added and then the

mixture was aged for 1 hour and filtered, washed, dried and calcined as previously described. For deposition-precipitation the Zn oxide was charged into the reaction vessel, $Pd(NO_3)_2$ solution was then added. The method then proceeded as before, with the precipitating agent added, the mixture aged for 1 hour and filtered, washed, dried and calcined as previously described.

1.3.2. Chemical vapour impregnation (CVI)

$Pd(acac)_2$ (99 %) and $Zn(acac)_2$ (99 %) were obtained from Sigma Aldrich. When used, the commercial ZnO support was supplied by Acros Organics (≥ 99.5 %) or by Sigma Aldrich (99.9%). The chemicals were used as provided and without further purification.

Bahruji *et al.* have previously proven CVI to be a successful method for producing Pd and PdZn catalysts for CO_2 hydrogenation to methanol.[3] The appropriate amount of $Pd(acac)_2$ was combined with the ZnO support in a glass vial and vigorously shaken for 30 seconds to 1 minute. The mixture was then tipped in to a Schlenk flask and sealed then lowered into an oil bath preheated to 80 °C. The Schlenk line was evacuated (~10^{-3} mbar) and the temperature increased to 133 °C and maintained for 1 hour. The product was then allowed to cool for 15 minutes under vacuum, then recovered from the Schlenk flask and calcined in static air for 16 hours at 500 °C. To produce PdZn/ZnO catalysts, the appropriate amount of $Zn(acac)_2$ was also added to the mixture of $Pd(acac)_2$ and ZnO in the glass vial.

1.3.3. Sol immobilisation

Polyvinyl alcohol (Mw = 10,000, 80 % hydrolysed), sodium borohydride (> 96 %), and zinc oxide (99.9 %) were all supplied by Sigma Aldrich. The chemicals were used as provided and without further purification.

For sol immobilised catalysts, palladium loading on ZnO 1 was 5 wt.%. Polyvinyl alcohol was used as a stabiliser and $NaBH_4$ as a reducing agent. An aqueous solution of $Pd(NO_3)_2$ of the desired concentration was prepared. Polyvinyl alcohol (PVA, 1 wt.% solution) was added to the stirring solution (PVA/Pd = 0.65 weight ratio). A 0.1 M freshly prepared $NaBH_4$ solution ($NaBH_4$/Pd = 5 molar ratio) was added to form a dark brown sol. After 30 min, the colloid was immobilised by adding ZnO 1. Concentrated sulphuric acid was added dropwise until pH 1-2 was reached. After 2 h, the slurry was filtered and washed with 2 L of deionised water before drying in air (110 °C, 16 h).

Table 1. A comparison between Pd/γAl_2O_3 and PdZn/ZnO (catalyst 1). The Pd was deposited on alumina (100 m^2g^{-1} surface area) by CVI

Sample	Conversion at 230 °C	Selectivity at 230 °C	Conversion at 270 °C	Selectivity at 270 °C
Pd/γAl_2O_3	2	17	6	6
PdZn/ZnO (catalyst 1)	8	58	18	25

2. Characterisation

2.1. X-ray diffraction

X-ray diffraction (XRD) was measured using Panalytical X'Pert Pro powder diffractometer (Malvern Panalytical, Malvern, UK) using a Cu source operated at 40 keV and 40 mA with a Ge(111) monochromator to select Kα1 X-rays. Patterns were analyzed from measurements taken over the 2θ angular range 10–80° (step size of 0.016°).

Figure S1. XRD patterns for catalysts 3, 4, 6, 7, 9 and 10 after reduction (400 °C, 1 h, 5 °C min^{-1}, 5 % H_2/Ar): a) wide scan, b) expanded scan.

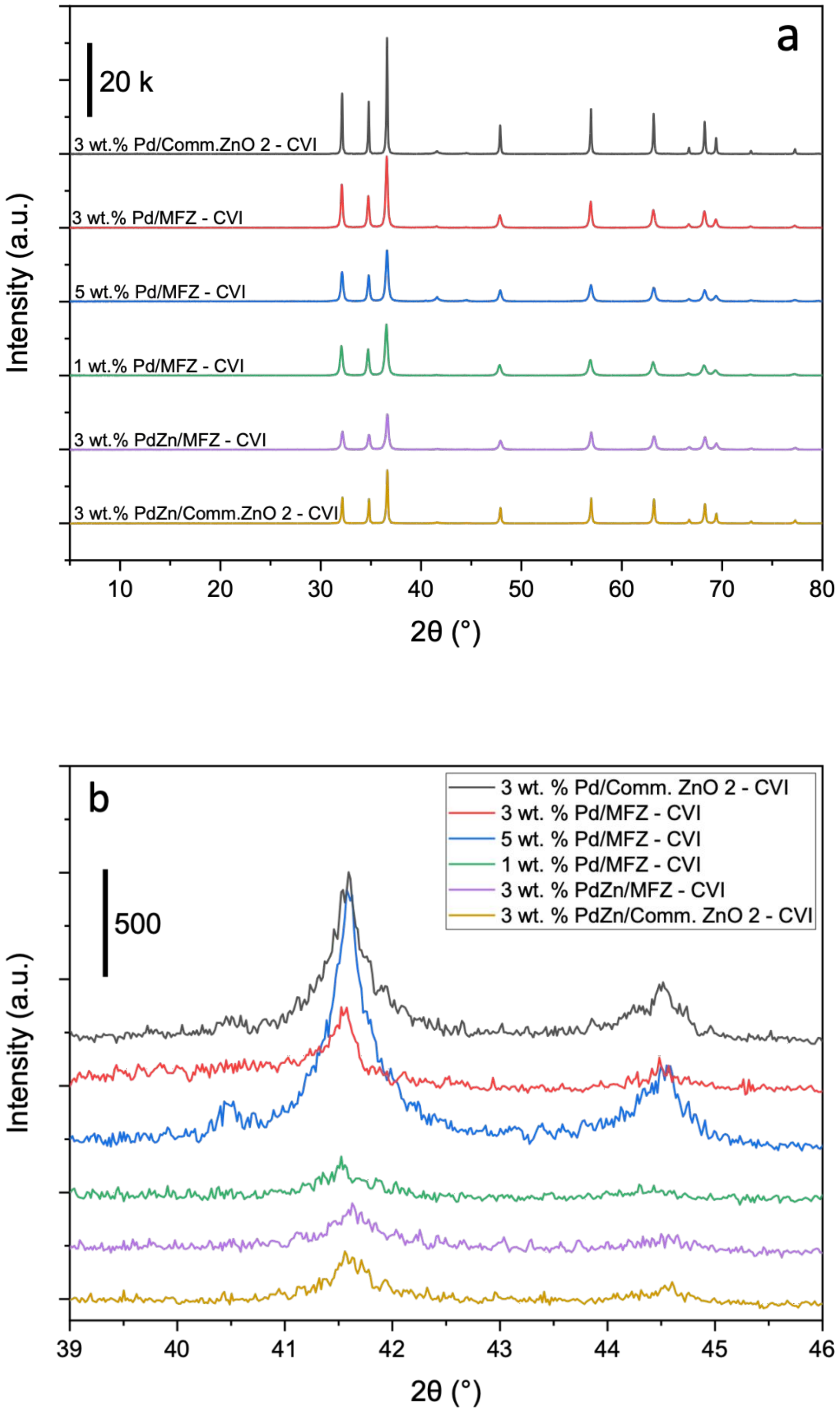
a
20 k
3 wt.% Pd/Comm.ZnO 2 - CVI
3 wt.% Pd/MFZ - CVI
5 wt.% Pd/MFZ - CVI
1 wt.% Pd/MFZ - CVI
3 wt.% PdZn/MFZ - CVI
3 wt.% PdZn/Comm.ZnO 2 - CVI
Intensity (a.u.)
2θ (°)
b
500
3 wt. % Pd/Comm. ZnO 2 - CVI
3 wt. % Pd/MFZ - CVI
5 wt. % Pd/MFZ - CVI
1 wt. % Pd/MFZ - CVI
3 wt. % PdZn/MFZ - CVI
3 wt. % PdZn/Comm. ZnO 2 - CVI
Intensity (a.u.)
2θ (°)

2.2. N_2 adsorption and desorption

Surface area was analysed using nitrogen adsorption at -196 °C and calculated by the Brunauer-Emmett-Teller (BET) method using a Quantachrome Nova 2200e. Prior to surface area analysis, the catalysts were degassed for 3 hours at 150 °C.

2.3. Scanning Transmission electron microscopy. Scanning transmission electron microscopy was performed using a JEOL ARM200F microscope at the electron Physical Sciences Imaging Centre (ePSIC) at Diamond Light Source. An acceleration voltage of 200 kV, a beam current of approximately 170 pA, a convergence semi-angle of 23 mrad and a HAADF inner angle of 80 mrad was used for data collection. EDX acquisition was performed using a JEOL Centurio dual-detector.

Figure S2. STEM-EDX data of 5 wt.% Pd/ZnO (catalyst 1) after reduction.

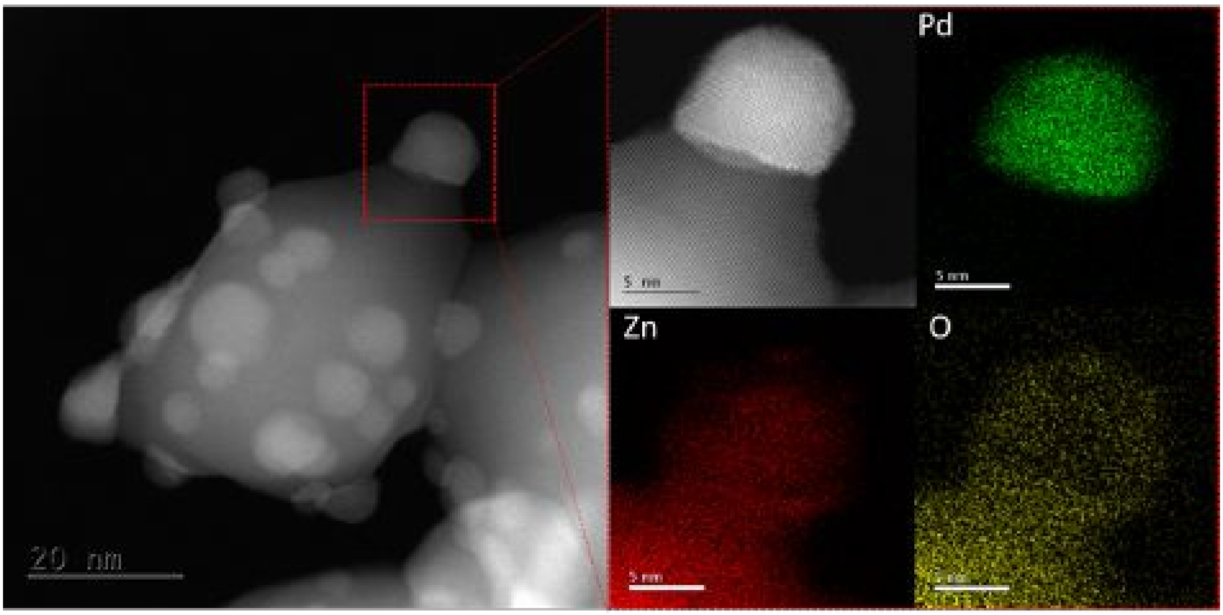

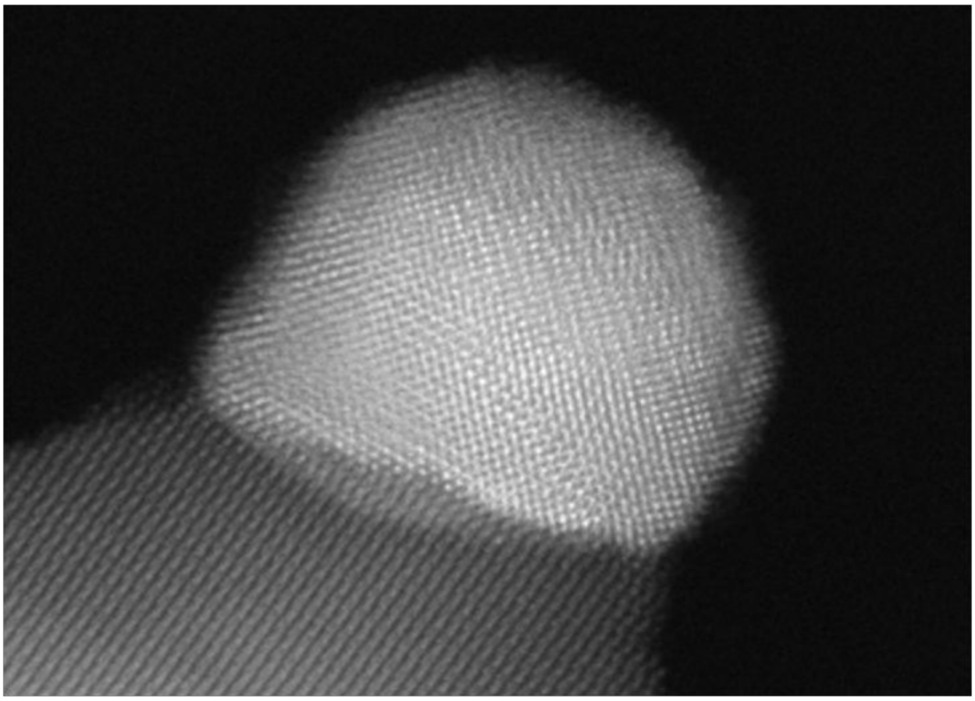

Figure S3. STEM images of 5 wt.% Pd/ZnO (catalyst 1) after calcination, but before reduction. There is no alloy formation and the Pd is present as PdO.

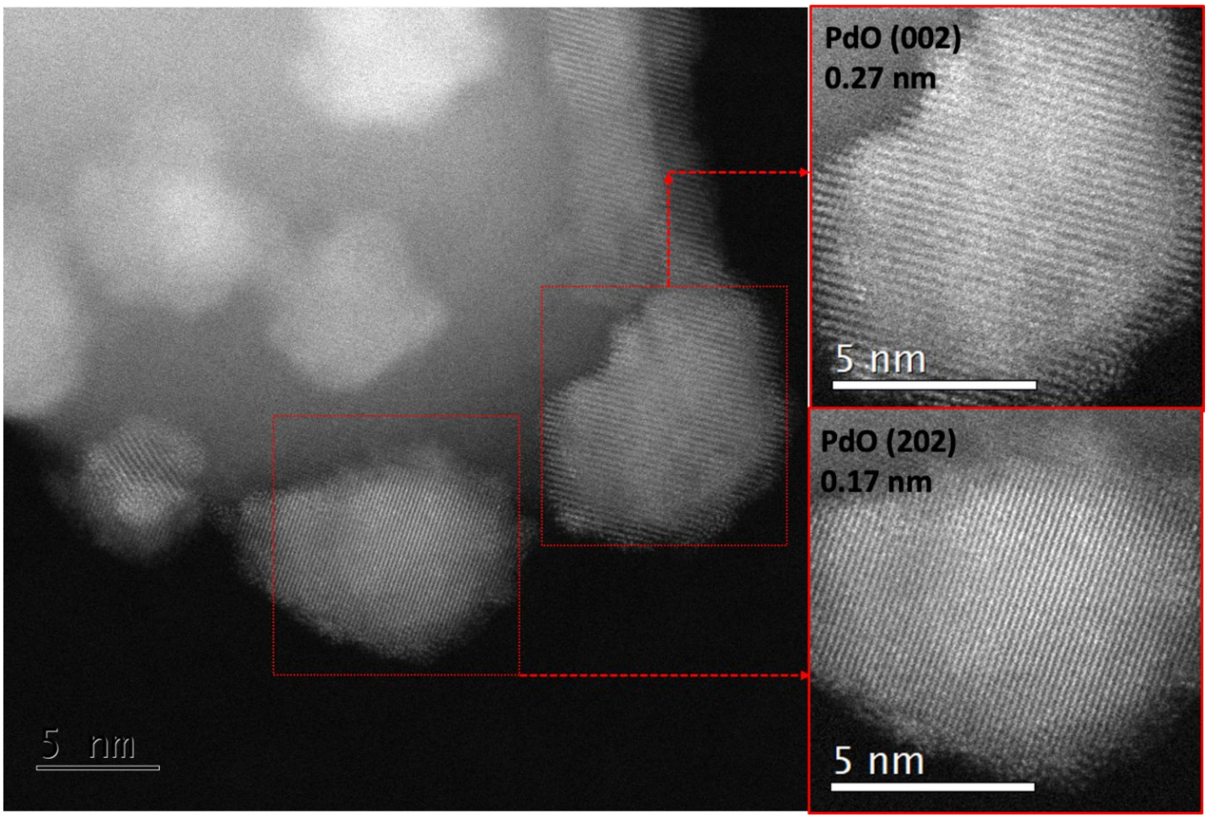

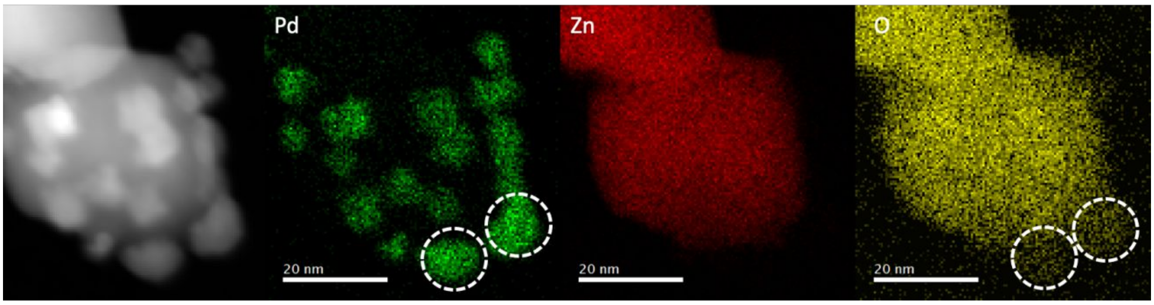

Figure S4

a) STEM images for sample 6, 5% Pd/MFZ, Pd deposited by CVI. The sample was reduced at 500 °C for 1 hour. b) the same, except reduced at 200 °C

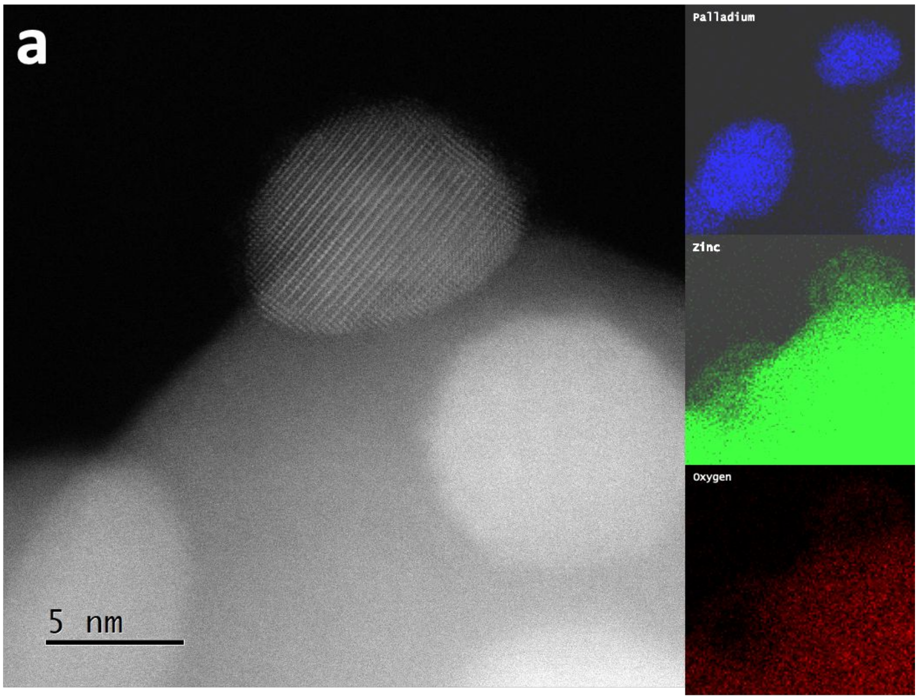

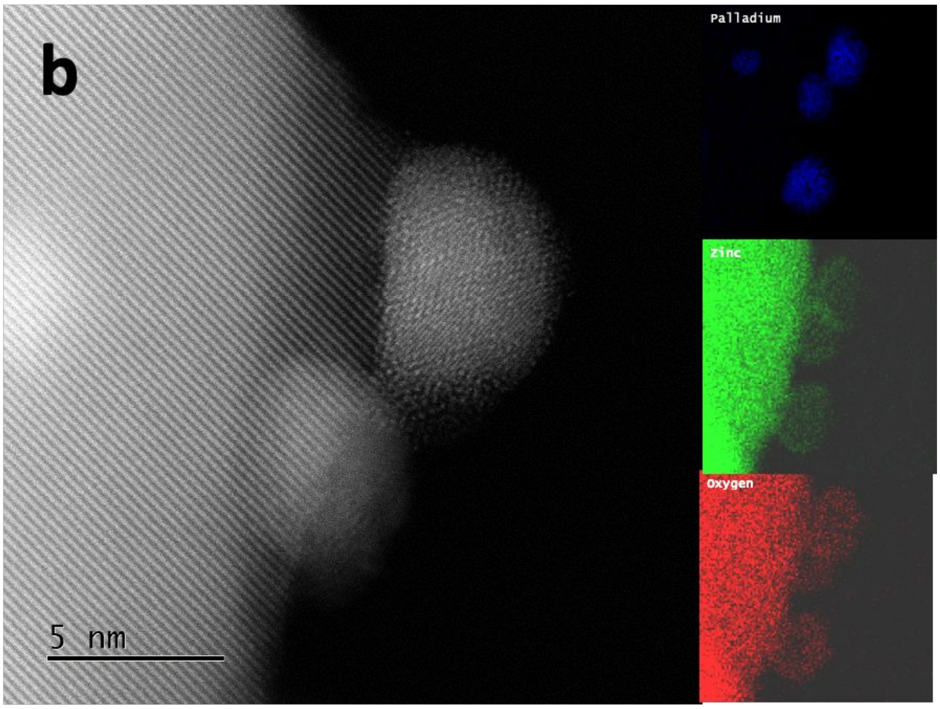

3. Reactor data. Here we give estimates of the uncertainties in the reactor data values for conversion and selectivity. Figure S5 shows data for one particular type of catalyst, prepared by author JRE, made by CVI of Pd onto Sigma Aldrich ZnO with 5% loading. The catalysts were made in two separate batches at different times and loaded in two different runs, into different beds of the 16-bed reactor. It can be seen that the mean deviation in conversion is ~0.5%, while for selectivity it is ~3% for the data at 230 °C (left panel), while at 270 °C it is ~0.6% for conversion and 0.8% for selectivity. In turn, each of these data points is taken from an average of four measurements over a period of 24 hours, where the mean deviation in conversion for a single sample was 1% at 230 °C and 0.6% at 270 °C, and in selectivity was 0.3% at each temperature.

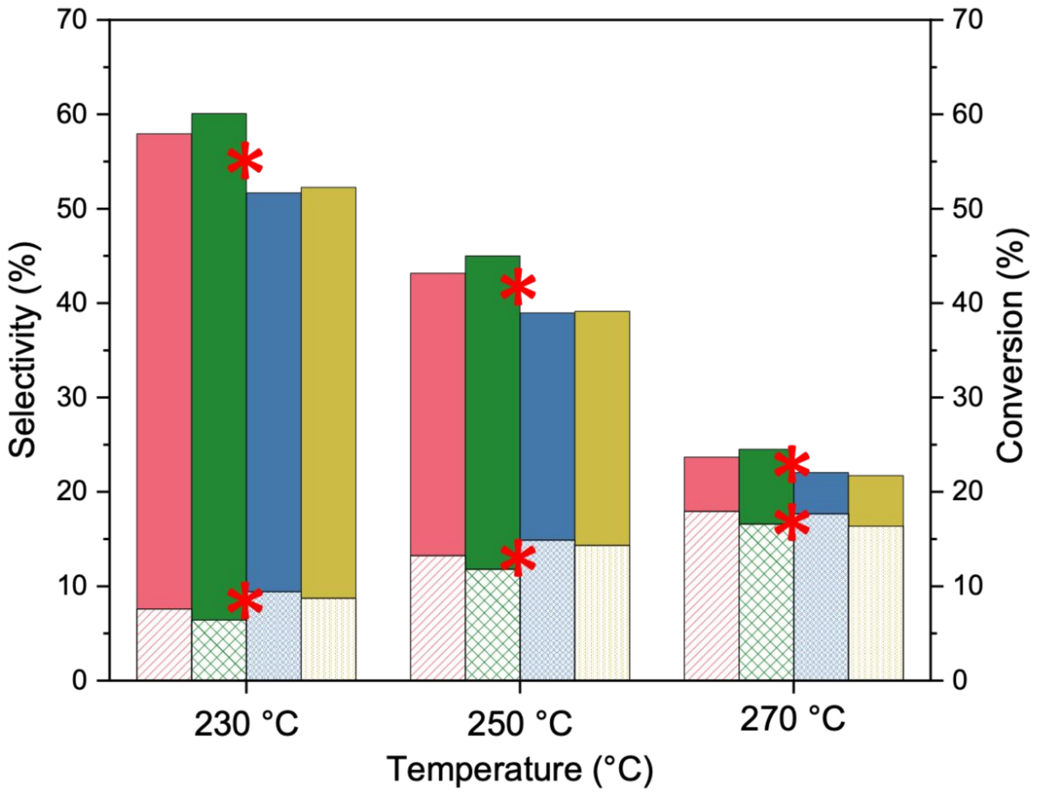

Figure S5. Bar graph for 5 wt.% Pd/ZnO, showing data for 4 catalysts, prepared in two separate batches, and run in different tubes, to give an idea of the mean deviation of data. The filled bars are methanol selectivity, while the hatched bars are conversion, the asterisks represent the averages of conversion and selectivity for the four catalysts.

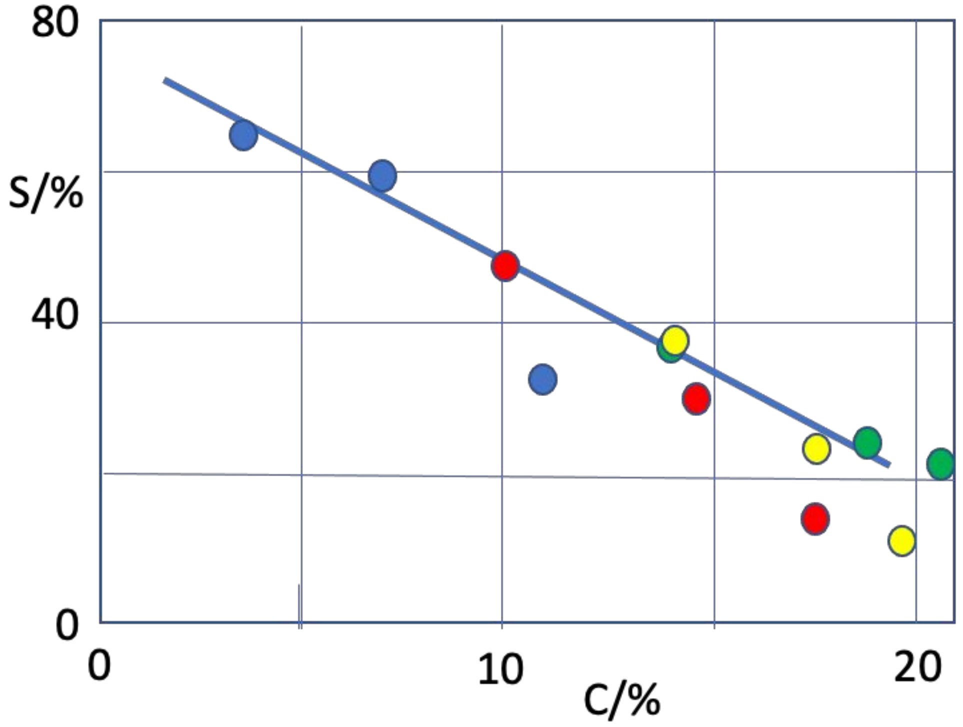

Figure S6. Selectivity-conversion dependence for several weight loadings of catalyst (15%Pd/MFZ). Blue circles, 0.1g; red circles.0.3 g; green, 0.5 g and yellow 0.7 g. There are three data points for each weight, measured at temperatures of 230, 250 and 270 °C.

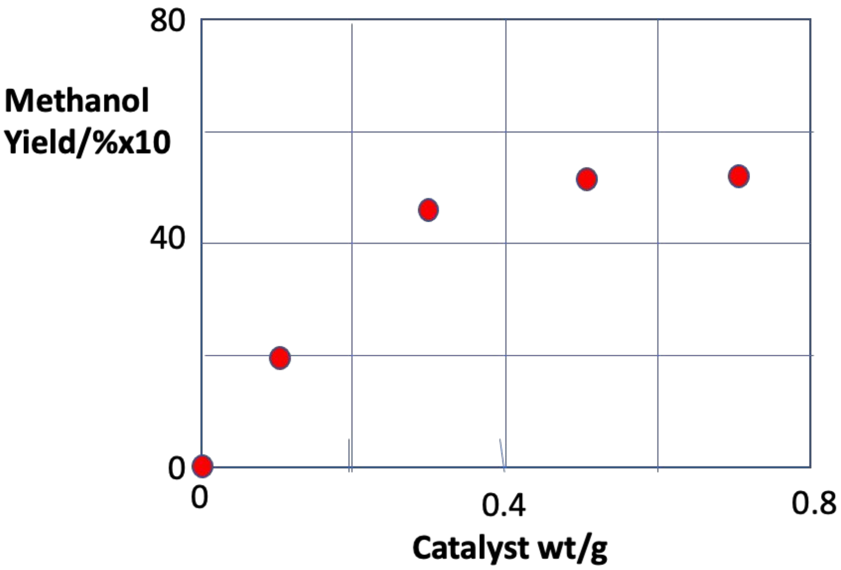

Figure S7. The weight loading dependence of the yield of methanol at 230°C, for the 15 wt. % Pd/Farag ZnO made by CVI of Pd.

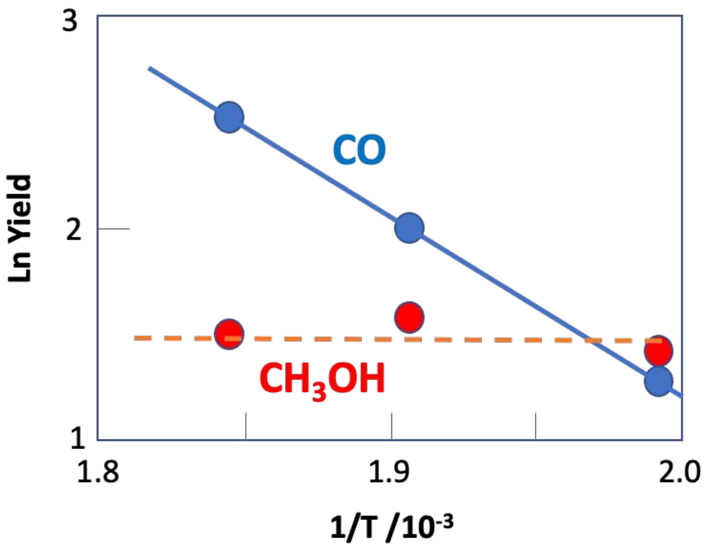

Figure S8. Arrhenius plot for CO and methanol production.

The turnover number was calculated as follows.

Using the data for catalyst 1 and the known average particle diameter, as shown in the main text, fig. 3 of the main text, of around 3.6nm. The metal surface area (MSA) then can be approximated by the following

$$MSA = 3W/\rho r$$

Where W is the weight of metal (g), ρ is its density (g. m^{-3}) and r is the average particle radius. Since we use the radius of the alloy particle, then we use an average density of 9.5 x 10^6 g m^{-3}, and the weight W of the metal particle must be more than that of the original Pd in the sample (0.025g) and is increased by the addition of equimolar amounts of Zn, adding to this weight by (65/106) x 0.075 g = 0.015 g, giving a total weight of metal particles of 0.04 g. This results in a surface area of 7 m^2. We do not know the exact number of sites per unit area, since the surface will be composed of a number of different exposed planes and steps etc, but we approximate this to 1x10^{19} sites m^{-2}. So, with that approximation in mind we have around 7 x 10^{19} surface metal sites.

The conditions for the data of fig. 1, sample 1, at 250 °C reaction temperature then conversion of CO2 is 13.5% giving 0.9 ml min^{-1} of CO_2 converted or 0.015 ml s^{-1}. If we now convert this to molecular units, then this equates to (0.015 ml s^{-1} x 6 x 10^{23} molecules mol^{-1} /24000 ml) which is 3.8 x 10^{17} molecules s^{-1}. The overall turnover frequency is then given by the following

Flow rate of converted CO_2/total sites = $3.8 \times 10^{17}/7 \times 10^{19} = 5.5 \times 10^{-3}$ molecules $site^{-1}$ s^{-1}. The turnover frequency to methanol, at a selectivity of 40% (fig. 1 of main text) is then 2.2×10^{-3} molecules $site^{-1}$ s^{-1}.

References

1. Farag, H. K., Hanafi, Z. M., Dawy, M., Abd, E. M., Aziz, E. Characterization of ZnO Nanopowders Synthesized by the Direct Precipitation Method. *Can. J. Pure Appl. Sci.* **4** 1303–1309 (2010).
2. Bowker, M., Houghton, H., Waugh, K. C., Giddings, T., Green, M., Crystal Plane Dependence of Adsorption and Reaction on Zinc Oxide. *J. Catal.* **84** 252–255 (1983).
3. Bahruji, H., Bowker, M., Jones, W., Hayward, J., Ruiz Esquius, J., Morgan, D. J., & Hutchings, G. J., PdZn Catalysts for CO2 Hydrogenation to Methanol Using Chemical Vapour Impregnation (CVI). *Faraday Discuss.* **197** 309–324 (2017).
